# Ray-Stretching Statistics and Hot-Spot Formation in Weak Random Disorder

**DOI:** 10.3390/e25010161

**Published:** 2023-01-13

**Authors:** Sicong Chen, Lev Kaplan

**Affiliations:** Department of Physics and Engineering Physics, Tulane University, New Orleans, LA 70118, USA

**Keywords:** random scattering, random potential, extreme event statistics

## Abstract

Weak scattering in a random disordered medium and the associated extreme-event statistics are of great interest in various physical contexts. Here, in the context of non-relativistic particle motion through a weakly correlated random potential, we show how extreme events in particle densities are strongly related to the stretching exponents, where the ’hot spots’ in the intensity profile correspond to minima in the stretching exponents. This strong connection is expected to be valid for different random potential distributions, as long as the disorder is correlated and weak, and is also expected to apply to other physical contexts, such as deep ocean waves.

## 1. Introduction

### 1.1. Rogue Waves

In the past few decades, many encounters with extreme oceanic waves have been publicized and documented. These historical records include a 25.6 m wave that hit the Draupner oil platform in the North Sea in 1995, two ships that suffered damage at 30 m above sea level from a single wave in the South Atlantic in 2001, and the cruise liner Norwegian Dawn that met a series of three 21-m waves off the coast of Georgia in 2005. Such waves, known as freak ocean waves, or rogue waves, are of extreme height relative to the typical wave in a given sea state. Satellite images taken in 2001 and analyzed as part of the European MaxWave project [1] detected ten waves above 25 m in height, suggesting that such waves commonly occur in the world’s oceans.

Freak waves have attracted much interest over the years, especially for quantitative predictions for freak wave probability distributions. Commonly known approaches to model freak waves include the Longuet–Higgins random sea model [2], the Nonlinear Schrödinger Equation (NLS) and its extension in the Dys equation [3,4], and ray dynamics in the work of White and Fornberg [5]. The Longuet–Higgins random sea model is based on random linear superposition of many plane waves with different directions and wavelengths, where (unlucky) constructive interference leads to an extreme event. By the central limit theorem, in this model the sea surface height at any spatial location behaves as a Gaussian random variable with some standard deviation σ. In the limit of a narrow frequency spectrum, the crest height then follows a Rayleigh distribution: the probability of crest height exceeding *H* is given by

(1) $$P_{\mathrm{Rayleigh}}\left(H\right)=e^{-H^{2}/2\sigma^{2}}.$$

According to observational data [1], this purely stochastic Rayleigh model significantly underestimates the actual probabilities of freak waves. There are also several alternative theories of the freak wave phenomenon [6].

The NLS or the Dysthe equation works well to incorporate nonlinear effects in the regime of small or moderate values of wave steepness kH, where *k* is the wave number. Since nonlinear effects scale as a power of the wave steepness, strong nonlinear evolution is more likely to come from initial conditions with already unusually high waves. In other words, the tail of the crest height distribution is likely to be influenced by linear triggering mechanisms, even if subsequent nonlinear development is also significant.

One model that combines such a linear triggering mechanism with nonlinear evolution is the focusing or refraction of incoming plane waves by random current eddies [5], the study of which was motivated by the fact that many freak waves have been observed in regions of strong currents. Whenever a focusing current is present, an incoming plane wave evolves into caustics or singularities with infinite ray density, which—when smoothed due to nonzero wavelength and/or nonzero spread in the initial angle or frequency—forms a repeated and reproducible branched flow pattern. Consequently, freak waves appear within the ’branches’ of enhanced intensity. Statistics regarding the distribution of crest heights can be obtained by combining the stochastic random seas picture (given by a local Rayleigh distribution) and the statistics of ray focusing in the presence of random currents.

Besides extreme waves in the ocean, extreme waves are known to occur in many other physical systems, governed by different equations of motion, where the waves or rays are scattered by a weak random potential. Examples of extreme waves and branched flow have been reported on a wide range of length scales, including the branching of electron flow [7,8,9,10,11]; amplification of tsunami waves [12,13,14]; branching of light traveling through a soap film [15]; and freak waves in optical [16,17], acoustic [18,19], and microwave propagation [20,21]. These systems share similarities in statistics and scaling relations, suggesting that a universal theory of scattering in weak random potentials may describe these different phenomena. Indeed, a search for universality in branched flows through potentials with differing correlation structures has obtained success [22,23]. The theory extends naturally to the case of an anisotropic scattering potential [24]. Very recently, a one-parameter model was shown to describe the behavior of classical branched flow in a time-dependent, one-dimensional random potential (equivalent to a time-independent two-dimensional potential for weak scattering), and a two-parameter phase diagram was shown to describe the corresponding quantum branched flow [25]. A recent overview of the theory and applications of branched flow appeared in reference [26].

The starting point of our work is similar to the model employed by White and Fornberg [5], as developed further in reference [27], so here we begin with a brief review of that model. The ray dynamics of deep-water surface gravity waves are governed by the dispersion relation:

(2) $$\omega \left(\vec{k},\vec{r}\right)=\sqrt{g|\vec{k}|}+\vec{k}\cdot \vec{U}\left(\vec{r}\right)\;,$$

where $\vec{U}\left(\vec{r}\right)$ is the time-independent current velocity of root mean square strength $u_{0}$ and spatial correlation length ξ. An incoming wave field undergoes weak scattering when $u_{0}\ll v$ (wave speed $\vec{v}=\partial \omega /\partial \vec{k}$) and the scattering angle scales as $u_{0}/v$ after one correlation length ξ in the forward direction. Eventually, singularities appear as local focusing of the manifold of initial conditions. The first cusp singularities or ’hot spots’ will appear after a median travel distance:

(3) $$y=L∼\xi {\left(u_{0}/v\right)}^{-2/3}\gg \xi \;,$$

and at that distance scale, the angular spread of trajectories due to scattering is characterized by $\delta \theta ∼{\left(u_{0}/v\right)}^{2/3}$. The dimensionless ’freak index’ γ that describes the strength of scattering is defined as $\gamma =\delta \theta /\Delta \theta ∼{\left(u_{0}/v\right)}^{2/3}/\Delta \theta$, where Δθ is the initial angular spread of trajectories [28].

Ray dynamics is applicable for understanding wave behavior in the semiclassical regime, kξ≪1, where the potential slowly varies on the scale of a wavelength. In that regime, the wave-height distribution may be obtained as a convolution of the Rayleigh distribution, Equation (1), with the distribution of classical ray densities, depending only on the freak index γ [27].

In the following, we apply analogous methods to the study of Schrödinger evolution in a two-dimensional random potential, where the ray limit is given by classical Newtonian mechanics. The choice of dispersion relation for weak scattering is not important, as the locations of the first generation of hot spots follow a universal scaling law across various dispersion relations. This will be further discussed in Ref. [29].

### 1.2. Density and Stretching Exponents

For ray dynamics in weak random disorder, the relationships between density distributions and the properties of the disorder have attracted much research interest, and the location of extreme densities has been the major focus. However, while a random superposition of waves as in the Longuet–Higgins model [2] corresponds to a swarm of trajectories, properties of the ray density have not been directly connected to statistics of the individual propagating ray trajectories. Therefore, we introduce the ’stretching exponent’—which in the case of parallel ray bundles in one dimension coincides with the rarefaction exponent introduced by Shaw and Heller [30]—as a way to quantify the degree of exponential convergence or divergence among nearby ray trajectories, and demonstrate a strong correlation between stretching exponents and the density. The region with the highest density is shown to correspond to the strongest focusing as measured by the stretching exponent distribution, and the strong correlation that first appears in this region is shown to persist everywhere further along the forward direction.

The strong negative correlation allows the density distribution to be studied from the perspective of the distribution of stretching exponents. Furthermore, the stretching exponents are directly connected with individual ray trajectories via the evolving monodromy matrices. The evolving monodromy matrix of an individual ray trajectory can be obtained from the second derivatives of the random disorder along the trajectory. The detailed mathematics are discussed in Section 2.

A relationship between extreme ray densities and minimal stretching exponents is consistent with intuitive expectations—indeed, if one considers the paraxial approximation and the case where the density at a given point is generated solely by one neighborhood of rays, then that density will simply be inversely proportional to the stretching factor. However, in Section 3, we also show that in the region of initial hot spot formation, the average stretching exponent becomes *negative*, and this is accompanied by a peak in the stretching exponent variance, or scintillation index. For a chaotic system with positive maximal Lyapunov exponent, a negative dip in the average stretching exponents is quite surprising and demands further investigation. Possible explanations involve the relationship between the statistics of the stretching exponent and the distribution of second derivatives of the disorder, which is further discussed in Ref. [31]. In any case, the non-monotonic behavior of the stretching-exponent statistics with distance greatly enhances the appearance of hot spots and rogue waves on distance scales given by Equation (3), beyond what would be expected based on the assumption of a simple exponential stretching with time.

## 2. Materials and Methods

In the following study, we focus on a simple example of a Hamiltonian system and establish a general connection between the ray-stretching exponents and intensity. Instead of ocean waves refracted by random currents, our model is based on a single-particle Schrödinger wave function (or, in the ray limit, a non-relativistic particle) scattered by a random potential field. In the region of weak potential or weak currents, despite the different dispersion relations, the statistics of the two models should resemble each other closely [27,29].

**Figure 1:**
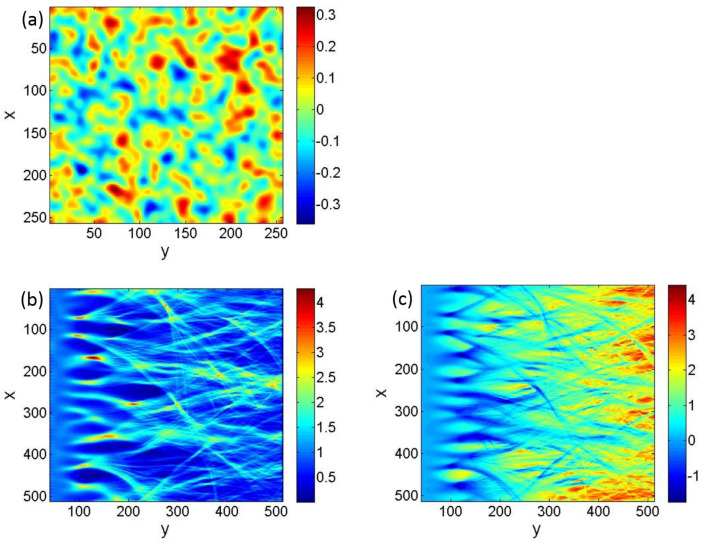
(**a**) A Gaussian random potential generated on a 256 by 256 grid with zero mean, standard deviation σ=0.1, and a Gaussian two-point correlation function with correlation length ξ=5. The potential is constructed by starting with ${\left(256\right)}^{2}$ delta-shaped peaks on a square grid with spacing unity, and then convolving with a Gaussian, as described in Equation (5). (**b**,**c**) The ray density and stretching exponent of particles scattered in the random potential field shown in (**a**), but with *x* and *y* rescaled to a 512 by 512 grid. Here (**b**) shows the particle density I(x,y), with intensity normalized to unity before scattering, and (**c**) shows the normalized average stretching exponent α(x,y). The correlation is clearly negative; i.e., higher densities in panel (**b**) are associated with a lower stretching exponent in panel (**c**).

A non-relativistic particle moving in a 2D potential follows classical Newtonian mechanics:

(4) $$\frac{d\vec{r}}{dt}=\vec{k},\;\frac{d\vec{k}}{dt}=-\frac{\partial \vec{V}\left(\vec{r}\right)}{\partial \vec{r}},$$

where energy $E=\frac{1}{2}{\left|\vec{k}\right|}^{2}+V\left(\vec{r}\right)$ (without loss of generality, taking ℏ=m=1) is conserved. In the following analysis, $V(\vec{r})$ is considered to be a random time-independent potential with zero average value, variance $\sigma^{2}=\bar{{V(\vec{r})}^{2}}$, and some two-point spatial correlation function $C(\vec{r}-{\vec{r}}^{\prime })$ characterized by correlation length ξ. In practice, the potential $V(\vec{r})$ may be constructed by a convolution between the shape of one potential bump $U(\vec{r})$ and a random noise $g(\vec{r})$:

(5) $$V∼F^{-1}\left[F\left[g\right]\cdot F\left[U\right]\right]\;,$$

and then normalized to have variance $\sigma^{2}$. Here $g\left(\vec{r}\right)=\sum_{i}h_{i}\delta \left(\vec{r_{i}}\right)$ (with $h_{i}$ random, independent, and having zero mean), F represents a Fourier transform, and the bump shape is related to the correlation function *C* as ${\left|F\left[U\right]\right|}^{2}∼F\left[C\right]$ [29]. A sample random potential constructed using ${\left(256\right)}^{2}$ Gaussian bumps is shown in Figure 1a.

The initial particles of total energy *E* are distributed uniformly in the *x* direction at $y=y_{0}$, with initial angles $\theta_{0}$ relative to the y direction drawn from a Gaussian distribution $P\left(\theta_{0}\right)∼e^{-{\theta_{0}}^{2}/2{\left(\Delta \theta \right)}^{2}}$, with a small but finite angular spread Δθ. (The choice of *y* as the propagation coordinate is consistent with the convention adopted, for example, in Ref. [27]). For the equations of motion (4), the scattering strength is determined by the dimensionless quantity σ/E in place of $u_{0}/v$. The particle trajectories will form a definite pattern of particle density for a given realization of the random potential field. Some regions (’hot spots’) will have above-average particle probability density, and others (’cold spots’) will have below-average density. Intuitively, the hot spots are more likely to be associated with local focusing of nearby particle trajectories, and the defocusing of trajectories will tend to produce cold spots.

To quantitatively monitor the degree of stretching or focusing, we define the stretching exponent α as the logarithm of the stretching ratio between nearby trajectories in the *x* direction, i.e., in the direction transverse to the main flow direction *y*:

(6) $$\alpha \left(t\right)=\log\left(\left|\frac{x_{1}\left(t\right)-x_{2}\left(t\right)}{x_{1}\left(0\right)-x_{2}\left(0\right)}\right|\right)=\log\left(\left|\frac{x_{1}\left(t\right)-x_{2}\left(t\right)}{\delta x(0)}\right|\right),$$

where trajectories 1 and 2 are initially parallel but separated by infinitesimal δx(0) in the *x* direction. The value of α(t) describes the cumulative divergence or convergence up to time *t*, and its time derivative dα(t)/dt gives the rate of exponential divergence or convergence at time *t*. The large-time limit of the stretching exponent in this 2D system is also the maximal Lyapunov exponent. While the concept of Lyapunov exponents is well established and studied in dynamical systems, the stretching exponent is employed here to study the behavior of chaotic systems for short or intermediate time scales (on scales around one Lyapunov time) and in a particular direction in phase space. For parallel ray bundles in one dimension, α coincides with the rarefaction exponent introduced by Shaw and Heller [30]. We are interested specifically in focusing or defocusing in the transverse position coordinate, which is most relevant for fluctuations in the position space density. Quantities of this type are widely applicable, for example, in semiclassical approximations, e.g., in the Van Vleck or Gutzwiller semiclassical propagator [32].

Very generally, dynamics in an *N*-dimensional space are described by the evolution of the phase-space vector: $\phi =\left(\phi^{\left[1\right]},\phi^{\left[2\right]},\ldots ,\phi^{\left[2N\right]}\right)=\left(r^{\left[1\right]},r^{\left[2\right]},\ldots ,r^{\left[N\right]},k^{\left[1\right]},k^{\left[2\right]},\ldots ,k^{\left[N\right]}\right)$; and the continuous flow may be considered as the limit of a discrete-time map where the time step approaches zero. The following analysis makes use of the discrete-time picture. Consider a map *F* from time step *n* to the next step n+1,

(7) $$\phi_{n+1}=F\left(\phi_{n}\right)\;.$$

The iteration of the tangent space is given by the Jacobian matrix:

(8) $$K_{ij}\left(\phi_{n}\right)=\frac{\partial F_{i}}{\partial \phi^{\left(j\right)}}{|}_{\phi =\phi_{n}}\;,$$

so that the shift $\delta_{n}$ in the phase vector $\phi_{n}$ is mapped to the next time step $K(\phi_{n})$ as

(9) $$\delta_{n+1}=K\left(\phi_{n}\right)\delta_{n}.$$

Therefore, the initial perturbation $\delta_{0}$ evolves by a product of Jacobian matrices to $\delta_{n}$ at time step *n*:

(10) $$\delta_{n}=M_{n}\delta_{0}=\left[K\left(\phi_{n-1}\right)K\left(\phi_{n-2}\right)\ldots K\left(\phi_{0}\right)\right]\delta_{0},$$

where $M_{n}$ is the monodromy or stability matrix. Similarly, in the continuous-time limit, dϕ/dt=f(ϕ), the stability matrix M(t) is given by dM(t)/dt=J(ϕ(t))M(t) where $J_{ij}=df_{i}/d\phi_{j}{|}_{\phi =\phi (t)}$ or dδ(t)/dt=J(ϕ(t))δ(t).

In the large-time limit, the 2N eigenvalues of *M* can be written in an exponential form: $\left\{e^{\lambda_{1}t},e^{\lambda_{2}t},\ldots ,e^{\lambda_{2N}t}\right\}$, where the spectrum of Lyapunov exponents $\left\{\lambda_{1},\lambda_{2},\ldots ,\lambda_{2N}\right\}$ is independent of time. The matrix *M* has several important properties. It generates a linear, canonical transformation, and the effective dimension of the spectrum is reduced from 2N to *N*, with a determinant equal to unity. Moreover, every independent constant of motion causes one pair of eigenvalues to become unity or one pair of exponents to vanish. Thus, in our 2D Hamiltonian model, with energy conserved, the eigenvalues of *M* are $\left\{e^{\lambda t},e^{-\lambda t},1,1\right\}$, where a single Lyapunov exponent λ completely captures the large-time behavior of the system.

On the other hand, the stretching exponent α defined by Equation (6) can also be viewed as

(11) $$\alpha \left(t\right)=\log(\left|M_{11}\left(t\right)\right|)\;,$$

where $\phi =\left(\begin{array}{c} x\\ y\\ k_{x}\\ k_{y} \end{array}\right)$, and for large time periods, the exponent α(t) is expected to grow linearly with time:

(12) α(t)=λt(t→∞).

However, the first generation of hot spots happens at intermediate time periods, $t∼\lambda^{-1}$, well before the stretching exponent α(t) begins to behave linearly. It is this intermediate behavior of α(t) that is of greatest interest for explaining the formation mechanism of the most extreme events.

The evolution of the displacement from time *t* to t+δt can be written out explicitly as

(13) $$\begin{array}{cc} \; & \delta {\left(\begin{array}{c} x\\ y\\ k_{x}\\ k_{y} \end{array}\right)}_{t+\delta t}=K\left(t\right)\cdot \delta {\left(\begin{array}{c} x\\ y\\ k_{x}\\ k_{y} \end{array}\right)}_{t}\\ \; & =\left[\begin{array}{cccc} 1-V_{xx}\delta t^{2} & -V_{xy}\delta t^{2} & \delta t & 0\\ -V_{xy}\delta t^{2} & 1-V_{yy}\delta t^{2} & 0 & \delta t\\ -V_{xx}\delta t & -V_{xy}\delta t & 1 & 0\\ -V_{xy}\delta t & -V_{yy}\delta t & 0 & 1 \end{array}\right]\cdot \delta \left(\begin{array}{c} x\\ y\\ k_{x}\\ k_{y} \end{array}\right), \end{array}$$

where $V_{xx}=\frac{\partial^{2}V}{\partial x^{2}}$, $V_{yy}=\frac{\partial^{2}V}{\partial y^{2}}$, and $V_{xy}=\frac{\partial^{2}V}{\partial x\partial y}$ are the second derivatives of the potential field V(x,y). Then, Equation (10) gives the monodromy matrix *M* at time *t* as M(t)=K(t−δt)K(t−2δt)…K(δt)K(0). For a specific trajectory, computing M(t) requires the positions of the particle at all the time points, which can only be obtained by integrating the equations of motion. However, upon ensemble averaging over homogeneous random potentials for a given potential variance and given a two-point potential correlation function, we can consider $V_{xx}$, $V_{yy}$, and $V_{xy}$ to be (correlated) random numbers drawn from appropriate distributions.

Moreover, when the initial angular spread Δθ in the forward *y* direction is small, Δθ≪1rad, the 2D model is analogous to a 1D model where the particle evolves in phase space (x,k) via a time-dependent random potential line V(x,t) with correlation time $\xi /\sqrt{2E}$. In the 1D model, the stretching exponent α is likewise defined via the exponential divergence of two trajectories with neighboring initial positions x(0) and x(0)+δx(0).

## 3. Results and Discussion

For numerically computing particle trajectories and stretching exponents in the 2D Hamiltonian model, we generated a Gaussian random potential $V(\vec{r})$ with Gaussian spatial correlations:

(14) $$\bar{V\left(\vec{r}\right)V\left({\vec{r}}^{\prime }\right)}∼C\left(\vec{r}-{\vec{r}}^{\prime }\right)=e^{-{\left(\vec{r}-{\vec{r}}^{\prime }\right)}^{2}/2\xi^{2}}\;,$$

by Fourier convolution, as in Equation (5), and normalized $V(\vec{r})$ to satisfy $\bar{V(\vec{r})}=0$ and $\bar{V^{2}\left(\vec{r}\right)}=\sigma^{2}$. The choice of a Gaussian-correlated random potential was made for convenience. The above theoretical discussion does not depend on any specific choice of a random ensemble, but only on the correlation length scale ξ and strength σ. The effect of varying the correlation function *C* is addressed in detail in Ref. [29].

The evolution was performed on a potential field of size 512 by 512 (in arbitrary units), with correlation length ξ=10 and a periodic boundary condition in the transverse (*x*) direction. A sample potential on a 256 by 256 grid, which was subsequently rescaled to a 512 by 512 grid for performing trajectory evolution, is shown in Figure 1a. The specific value of ξ was arbitrary and served merely to set the scale for the simulation. Without loss of generality, we set E=1 (energy). The strength of scattering γ can be controlled either by varying the potential strength, σ, or by controlling the angular spread, Δθ, in the initial conditions. To avoid boundary effects, particles were launched from $y_{0}=40$ inside the potential field and uniformly distributed in the transverse *x* direction, with initial angles $\theta_{0}$. The initial phase-space vector for each trajectory was $\left(x,y_{0},v_{0}\sin\theta_{0},v_{0}\cos\theta_{0}\right)$, for which the initial velocity $v_{0}$ was calculated based on its starting position $v_{0}=\sqrt{\frac{2(E-V\left(x,y_{0}\right))}{m}}=\sqrt{2(1-V\left(x,y_{0}\right))}$ so that energy was fixed (E=1) for all trajectories.

Each trajectory was evolved by integrating the equations of motion, Equation (4), using a fourth order Runge–Kutta integration method. Cubic interpolation was used for the potential $V(\vec{r})$ when running trajectories. The trajectories were weighted by the angular spread $P\left(\theta_{0}\right)∼e^{-{\theta_{0}}^{2}/2{\left(\Delta \theta \right)}^{2}}$, and then points along each trajectory were binned using Gaussian-shaped windows of size $\tilde{\xi }$ to generate a ray density map I(x,y). Gaussian-shaped binning eliminates any artificial discontinuity in the binned density and effectively smooths the density data I(x,y) on the scale $\tilde{\xi }$, which must be chosen to be small compared to the physical correlation scale ξ. A spacing of Δx=2 in the initial trajectory positions *x*, an increment of $1^{\circ }$ in the initial angle $\theta_{0}$, and Gaussian intensity bins of width $\tilde{\xi }=1$ with spacing 2 on the 512 by 512 grid were seen to be sufficient to achieve convergence in all the density data. Using initial positions $0\leq x_{0}<512$ with spacing Δx=2 and initial angles $-15^{\circ }\leq \theta_{0}\leq 15^{\circ }$ with spacing $\Delta \theta =1^{\circ }$ requires 7936 trajectories; see Figure 1b. For stronger scattering (larger γ), structures appear at a smaller scale so that the convergence of the density data requires a greater number of trajectories.

A typical density map for initial angular spread $\Delta \theta =5^{\circ }$ with a potential of strength σ=0.1 is shown in Figure 1b, where the first generation of caustics forms around 80<y<160, and the corresponding freak index is γ=2.5. Note that the particle density I(x,y) is normalized to unity, I(x,y)=1, before scattering.

Next, we demonstrate the connection between the density and the stretching exponent α(t). For every original trajectory launched at $\left(x,y_{0}\right)$, we launched a ’twin’ trajectory in the same potential at $\left(x+\delta x\left(0\right),y_{0}\right)$. Then, α(t) was computed for each trajectory according to Equation (6); the trajectories were weighted by the initial angular spread $P\left(\theta_{0}\right)∼e^{-{\theta_{0}}^{2}/2{\left(\Delta \theta \right)}^{2}}$; and the stretching exponents were eventually mapped into the same grid that was used for density data I(x,y) to produce an average position-dependent stretching rate α(x,y). Again, the binning was performed using a Gaussian window function, with a width chosen appropriately for data smoothing. Due to the long-term exponential stretching trend (Equation (12)), the initial separation δx(0) must be chosen sufficiently small so that the separation between twin trajectories remains small during the whole time evolution. In the following, we used $\delta x\left(0\right)=10^{-5}$. We have confirmed that our results are independent of δx(0) as long as $\epsilon_{m}\ll \delta x\left(0\right)\ll \xi$, where ξ=10 is the correlation scale of the potential and $\epsilon_{m}$ represents machine precision.

First, we notice the obvious connection between the density map Figure 1b and the stretching exponent map Figure 1c. The correlation is clearly negative; i.e., higher densities in Figure 1b are associated with a lower stretching exponent in Figure 1c. Indeed, every major hot spot (maximum) of the density map corresponds visually to a minimum at the same location in the stretching exponent map. This is consistent with our prediction that extreme density events would occur where the trajectories focus most significantly. Indeed, as noted above in Section 1.2, if we assume the paraxial approximation and further assume that the density at any given point (x,y) is all coming from parallel rays originating in the neighborhood of one initial point $\left(x_{0},y_{0}\right)$, then the proportionality $I\left(x,y\right)∼1/M=e^{-\alpha }$ will hold exactly. Of course, in reality, chaotic dynamics leads at sufficient time scales to caustics and folds in the time evolution, so that the density I(x,y) is given by a sum of contributions originating at different initial points with different exponents α.

**Figure 2:**
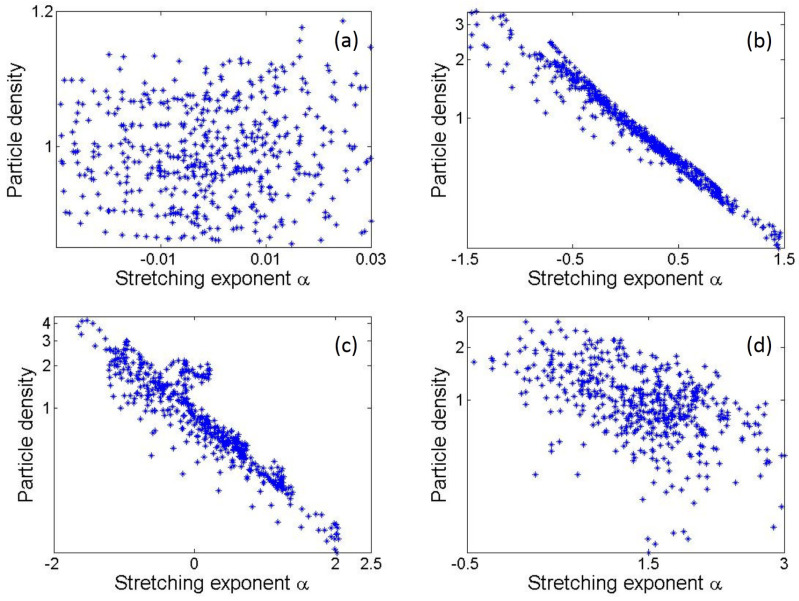
Scatter plots of density vs. stretching exponent before, during, and after the first generation of hot spots (around *y* = 80–130). (**a**–**d**) represent data for y=50 (before), y=100 (during), y=125 (during), and y=400 (after), respectively.

In Figure 2, we show scatter plots of the relationship between I(x,y) and α(x,y) before (y=50), during (y=100 and y=125), and after (y=400) the region with the strongest density fluctuations. Clearly, the stretching exponent is negatively correlated with density: The strong correlation grows as the rays encounter the first generation of caustics and eventually dies off after a few Lyapunov lengths. In the region of the first caustics, extremely high densities are always associated with most negative stretching exponents, and the largest stretching exponents lead to the lowest density levels. More specifically, the particle density scales as

(15) $$I\left(x,y\right)∼e^{-b\alpha (x,y)},$$

with constant coefficient *b* around the first caustics, as seen in Figure 2b,c. This relationship relies only on ray dynamics, and apart from the coefficient *b*, it does not depend on any specific dispersion relation. At larger time scales ($t\gg \lambda^{-1}$), the stretching exponents α grow linearly with time, as described by Equation (12), and the density probability distribution gradually collapses to a Gaussian one. In this regime, the correlation between the intensity and the stretching exponent declines and eventually disappears. The crossover to the large-time regime is illustrated in Figure 2d. Nevertheless, in the regime of greatest interest, i.e., in the first caustic region $t∼\lambda^{-1}$ where the strongest hot spots are present, the relationship is very robust.

**Figure 3:**
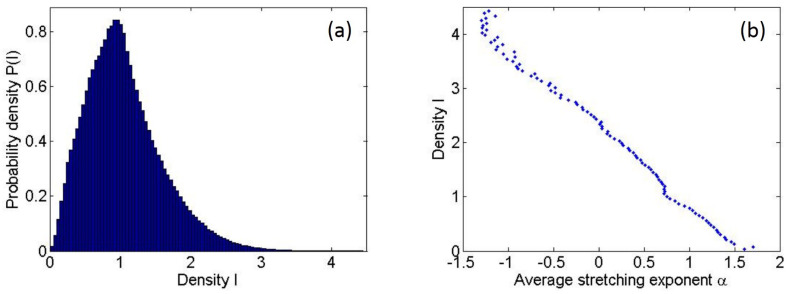
(**a**) Probability distribution of particle density *I* in the region 60<y<500. (**b**) Average stretching exponent α in the same region, with each data point corresponding to one bin in panel (**a**).

The distribution of the ray density is shown in Figure 3a, where all the density data points in the region 60<y<500 from five realizations of the ensemble are included (while the trajectories are launched at y=40 and the computational area extends through y=512, a slightly smaller region was used for collecting statistics to avoid possible edge effects). As mentioned earlier, the bulk of the distribution is close to a Gaussian distribution, whereas the fatter tail represents events of extreme densities. Then, for Figure 3b, we averaged the stretching exponents for all the spatial cells whose density values fall within each bin in Figure 3a. Not only in the area of the first caustics but the density distribution I(x,y) and stretching exponent distribution α(x,y) in general have a strong negative correlation; the locations with smaller or more negative stretching exponents are very likely to exhibit higher ray densities. Note that the fluctuations after very modest ensemble averaging in Figure 3b are remarkably small compared to fluctuations for one realization in Figure 2.

**Figure 4:**
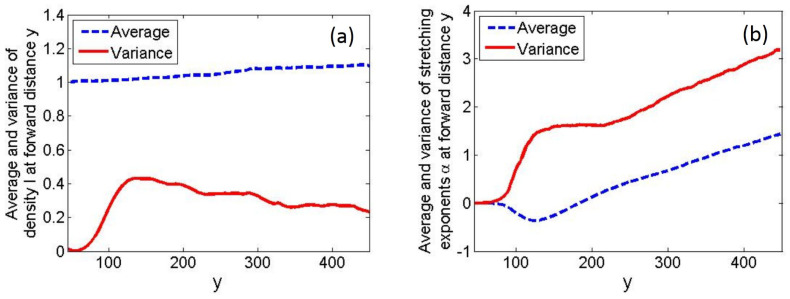
Evolution of intensity and stretching-exponent statistics as a function of forward travel distance. (**a**) Average and variance of the density I(x,y) at forward travel distance *y*. (**b**) Average and variance of the stretching exponent α(x,y) at forward travel distance *y*.

To further investigate the relationship between stretching exponent and ray density, we show in Figure 4 the average and variance of these two quantities as functions of the forward distance *y*. Here, for each value of *y*, we collected data over all transverse positions *x* and again used five different realizations of the random potential to reduce statistical noise.

The average density $\bar{I}\left(y\right)$ increases with the factor $\bar{{\left(\cos\theta \right)}^{-1}}$ as required by probability conservation of the ray dynamics, where θ is the angle measured from the forward *y* direction. In the region of small angular spread, $\bar{I}\left(y\right)∼\bar{{\left(\cos\theta \right)}^{-1}}\approx 1+\bar{\theta^{2}}/2=1+{\left(\Delta \theta \right)}^{2}/2+{\left(\delta \theta \right)}^{2}/2$, where ${\left(\Delta \theta \right)}^{2}$ and ${\left(\delta \theta \right)}^{2}$ are the variances associated with the initial angular spread and the scattering, respectively. As ${\left(\delta \theta \right)}^{2}$ increases linearly with forward distance *y*, the average intensity grows linearly with the forward distance, as observed in Figure 4a.

We now turn to the stretching exponent. In the regime of small-angle scattering in the forward *y* direction, we have $y\approx v_{0}t$, and the stretching exponent $\bar{\alpha }\left(y\right)$ averaged over the transverse direction must grow linearly with the forward distance *y* at large distances, in accordance with Equation (12), with slope $\lambda /v_{0}$. Furthermore, at large time scales, we can consider the process of focusing or defocusing in the transverse direction as a random walk or diffusive process, which gives rise to fluctuations in the stretching exponent α around its average value. Thus, the variance in the stretching exponents is also expected to grow linearly at large distances. The linear growth in both the average stretching exponent and its variance is observed in Figure 4b.

Of greater interest, however, is the behavior on the scale of a Lyapunov length $v_{0}/\lambda$, corresponding to 100<y<150 in Figure 4b. The marked dip in the average exponent $\bar{\alpha }\left(y\right)$ at short time scales indicates substantial local focusing, which is consistent with the visual evidence in Figure 1b. In this same region, we see in Figure 4b strong fluctuations in the stretching exponent (as measured by the variance), and correspondingly, in Figure 4a, large fluctuations in the ray density associated with the formation of the first and strongest hot spots. Here, we note that the density variance is closely related to the scintillation index, defined as $Var\left(I\left(y\right)\right)/{\left(\bar{I}\left(y\right)\right)}^{2}$, and in our case, $\bar{I}\left(y\right)\approx 1$ throughout. Subsequently, the variance in the density and the scintillation index decline as the number of independent trajectories contributing to the intensity at a given point grows exponentially when *y* is larger than a Lyapunov length, gradually washing out the pattern of hot spots and cold spots associated with extreme events.

The negativity of the average exponent $\bar{\alpha }$ at short time scales is rather surprising. This unexpected behavior can be confirmed analytically using perturbation theory over a small *t*, where it turns out that $\bar{\alpha }$ scales as $t^{3}$, with a prefactor depending on the correlation function of the random potential [29].

As the most significant fluctuations in both density and the stretching exponent were detected in the same spatial region, we confirm that local focusing of trajectories is directly correlated with the extreme high densities. Future work [29] extends the scaling relationship of the form (3) based on the scaling of the stretching-exponent statistics.

## 4. Conclusions and Outlook

We have seen that scattering of non-relativistic particles in a random weak potential field generates density patterns very similar to those observed for deep water ocean waves in the work of White and Fornberg [5]. The similarity in the intensity distributions in different physical contexts suggests the value of a universal perspective on the topic of scattering or refraction in a weak random disorder. Consequently, we explored the general connection between the density and stretching exponent in the context of non-relativistic particle motion. We conclude that the stretching exponent directly correlates with the density, and the negative correlation arises everywhere, including in the region of the strongest density fluctuations, and also further along the forward direction. By explicitly connecting these two quantities, we can treat the stretching exponent as a quantitative mirror for the intensity.

We also note that particle dynamics in a 2D ray model is analogous to a 1D model with a time-dependent potential in the regime of small angular spread. Whereas the full 4 by 4 monodromy matrix in the 2D model may be challenging for numerical evolution and even more so when it comes to an analytical treatment, the 1D model may be simple enough to obtain quantitative predictions for the distribution of the stretching exponent. Therefore, we are able to further explore the mechanism and statistics of freak wave events by studying monodromy matrix statistics in the 1D model [29].

Our calculations here are based on a model where nonlinear effects are absent. The linear model may be regarded a starting point for a more sophisticated nonlinear analysis. Nevertheless, nonlinear wave effects become significant only where the intensity produced by linear mechanisms is already large. It is therefore unlikely that nonlinear effects would substantially change the strong negative correlation between the density and stretching exponent.

While the long time behavior of the stretching exponent displays simple linear growth, the behavior on the scale of the Lyapunov time is of greater interest due to the appearance of the first generation of hot spots. A surprising minimum in the average stretching exponent is seen to correspond to a peak in the intensity variance in this regime. Future work [29] will investigate in much greater detail the statistics of the stretching exponent as a function of system parameters and distance from the origin, and explore the robustness of the stretching-exponent statistics with respect to changes in the equations of motion and the correlation properties of the random scattering potential.

## Data Availability

Data available from the authors on request.
